# A Network Pharmacology Approach to Investigate the Mechanism of Erjing Prescription in Type 2 Diabetes

**DOI:** 10.1155/2021/9933236

**Published:** 2021-07-24

**Authors:** Jiexin Wang, Haiqing Chu, Hangying Li, Wenqian Yang, Yu Zhao, Tong Shen, John Cary, Liming Zhang

**Affiliations:** ^1^College of Pharmacy, Ningxia Medical University, Yinchuan 750004, China; ^2^Ningxia Engineering and Technology Research Center for Modernization of Chinese Medicine, Yinchuan 750004, China; ^3^Ningxia Medical University, Yinchuan 750004, China; ^4^Key Laboratory of Ningxia Ethnomedicine Modernization, Ministry of Education, Ningxia Medical University, Yinchuan 750004, China

## Abstract

Erjing prescription (EJP) was an ancient formula that was recorded in the General Medical Collection of Royal Benevolence of the Song Dynasty. It has been frequently used to treat type 2 diabetes mellitus (T2DM) in the long history of China. The formula consists of *Lycium barbarum* L. and *Polygonatum sibiricum* F. Delaroche with a ratio of 1 : 1. This study aimed to identify the potential effects and mechanisms of EJP treatment T2DM. The target proteins and possible pathways of EJP in T2DM treatment were investigated by the approach of network pharmacology and real-time PCR (RT-PCR). 99 diabetes-related proteins were regulated by 56 bioactive constituents in EJP in 26 signal pathways by Cytoscape determination. According to GO analysis, 606 genes entries have been enriched. The PPI network suggested that AKT1, EGF, EGFR, MAPK1, and GSK3*β* proteins were core genes. Among the 26 signal pathways, the PI3K-AKT signal pathway was tested by the RT-PCR. The expression level of PI3K p85, AKT1, GSK3*β*, and Myc mRNA of this pathway was regulated by EJP. The study based on network pharmacology and RT-PCR analysis revealed that the blood sugar level was regulated by EJP via regulating the PI3K-AKT signal pathway. Plenty of new treatment methods for T2DM using EJP were provided by network pharmacology analysis.

## 1. Introduction

Type 2 diabetes (T2DM, Chinese name: Xiaoke) is a common chronic metabolic disease with a high prevalence. In 2019, over 463 million people suffered from diabetes. It is estimated that there will be 578 and 700 million diabetic patients in 2030 and 2045, respectively [1, 2], which would bring up a huge economic burden on society. In general, many cases may be undiagnosed in a long predetection period [3]. A lot of complications, such as heart failure, cardiovascular complications, and diabetic nephropathy, are always accompanied with the occurrence of diabetes [4–7]. Low immunity is a common feature for almost all forms of diabetes [8]. Therefore, the researcher devotes to exploring effective drugs to prevent and treat T2DM. Nowadays, traditional Chinese medicine (TCM) has been increasingly popular for the T2DM treatment [9]. For example, Liuwei Dihuang pills and Huanglian decoction have been widely researched for treating T2DM [10–12]. Alizarin, isolated from TCM (*Rubia cordifolia*), reduced blood sugar levels and alleviated insulin resistance through the PI3K/Akt pathway [13].

EJP (also known as Erjingwan) is a formula consisting of *Lycium barbarum* L. and *Polygonatum sibiricum* F. Delaroche, which is recorded in General Medical Collection of Royal Benevolence, early traced back to the Song Dynasty. Erjingwan, a classic formula of nourishing, showed antiaging effects in the skin through activation of Nrf2 and inhibition of NF-*κ*B [14]. These two medicines possessed a certain hypoglycemic effect and mainly contained polysaccharides and flavonoids [15]. The extract of EJP exhibited hypoglycemic and hypolipidemic activities on the model mouse of T2DM [16]. *L. barbarum* had a substantial value as a homology of medicine and food [17]. Rui Zhao reported *L. barbarum* polysaccharides improved the insulin resistance in NIDDM rats [18]. Galactomannan extracted from Iraqi *L. barbarum* fruit treatment improved the bodyweight and alleviated diabetic complications associated with lipid peroxidation in the alloxan-induced diabetes in rats [19]. Besides, phenolic amides from *L. barbarum* also had an immunomodulatory activity [20]. It was recorded that *P. sibiricum* had a rich pharmacological function, such as strengthen immunity, cardioprotection, antibacterial, and antidiabetes [21], in modern and ancient pharmacology literatures. *P. sibiricum* polysaccharide improves inflammatory cytokines and promotes glucose uptake in high glucose and high insulin-induced 3T3-L1 adipocytes by promoting Nrf2 expression [22]. Although EJP had been applied to treat T2DM, the mechanism was not completely elucidated.

In this study, network pharmacology can be used to elucidate the correlation between components (except for the *L. barbarum* polysaccharide) and pathways by constructing a network model. First, effective components from EJP, certain targets of these components, and the gene of T2DM were excavated through the TCMSP database. Second, the interaction of compound and proteins were elucidated through the network models of compound-target and key proteins. The network of the component-target-pathway was also built to analyze the mechanism of T2DM treated by EJP. Third, the PI3K p85, AKT1, GSK3*β*, and Myc mRNA of PI3K-AKT signal pathways were validated at the RT-PCR analysis. In total, the research will be expected to provide new strategy and thinking on the study for EJP.

## 2. Materials and Methods

### 2.1. The Preparation of Aqueous Extract from EJP

The *Lycium barbarum* L. were purchased from Ningxia, PR China (batch no. 070101, 104.17°–107.39°E, 35.14°–39.23°N), and *Polygonatum sibiricum* F. Delaroche were purchased from Sichuan, PR China (batch no. 070101, 97.21°–108.31°E, 26.03°–34.19°N). *L. barbarum* and *P. sibiricum* were dried severally and extracted (1 h × 3) with water at 1 : 1 ratio. Then, the aqueous extract was concentrated and dried. After that, the extract with a concentration of 1 mg/mL was prepared to treat IR-HepG2.

### 2.2. Ingredients Database Construction

All potential compounds of EJP were obtained from the Traditional Chinese Medicine Systems Pharmacology (TCMSP) database analysis platform database (http://lsp.nwu.edu.cn/tcmsp.php/) [23]. In the TCMSP database, 500 Chinese herbal medicines and 30,069 constituents were registered from the Chinese Pharmacopoeia (2010 edition). A total of 56 compounds were gained from EJP, including 45 in *L. barbarum* and 12 in *P. sibiricum*. The details of each class of compounds are summarized in Table 1.

First, effective constituents that contributed to its efficacy were selected by absorption, distribution, metabolism, and excretion parameters (ADME), meanwhile those are ineffective or even toxic were removed [24]. Second, higher oral absorption, bioavailability, and biological properties were essential for candidate constituents. Therefore, these compounds needed to satisfy the 30% in oral bioavailability (OB) and 0.18 in drug-likeness (DL).

The related proteins of active components in EJP were acquired from TCMSP databases. Genes related to proteins were retrieved from the UniProt Knowledgebase (UniProtKB) (http://www.uniprot.org), which was a protein database containing 54,247,468 sequence entries [25].

### 2.3. Predicting Targets of T2DM

The target proteins of T2DM were obtained from four sources: (1) 15,500 gene entries were included in OMIM database (http://www.omim.org/), which was focused on illustrating gene and genetic disorders [26]. (2) GeneCards (https://www.genecards.org/) is a comprehensive database that provided human genes of annotation and prediction. It could effectively establish the linkages of gene disease [27]. (3) Dis-GeNET (http://www.disgenet.org/) database, the current release contains more than 24,000 diseases and traits, 17,000 genes, and 117,000 genomic variants [28]. (4) Therapeutic target database (TTD) contained information from three aspects: (i) the microRNAs and transcription factors of target regulation, (ii) the proteins of target interaction, and (iii) targeting agents and targets, which can be easily retrieved and further enriched by the mechanisms of regulation or biochemical classes [29]. All targets linked to T2DM were only limited to *Homo sapiens*. The 1,260 genes totally were gained. The 99 same target proteins of compound and disease were selected as the main target of EJP in the treatment of T2DM (Table 2).

### 2.4. Network Construction

#### 2.4.1. Compound-Target Network

The pharmacological mechanisms of action were explored by the compound-target network, which was founded for the 57 compounds and relevant protein targets in T2DM utilizing Cytoscape 3.7.2. [30].

#### 2.4.2. GO and KEGG Analyses

Gene ontology (GO) is to annotate genes and their expression products. It is mainly divided into 3 parts: cell component (CC), biological process (BP), and molecular function (MF) [31]. Kyoto Encyclopedia of Genes and Genomes (KEGG) can analyze the signal pathways of drug targets in order to search the disease signal pathways that have maximum correlation, which is significant for discovering the possible mechanism of EJP in the treatment of T2DM [32]. In this study, the enrichment analysis of GO and KEGG were performed through DAVID bioinformatics resources (https://david.ncifcrf.gov) [33] in order to explore the related CC, BP, MF, and pathways. It illustrated the connection of genes and target proteins in diabetes.

#### 2.4.3. PPI Network

The effective components of drugs effected on the human body not only by directly acting on one target but also by indirectly acting on other targets. The prevention and control disease was regulated by multifarious intricate signal pathways, which are interactions and transductions between upstream and downstream targets. The complex relationship between targets and targets can be clearly displayed by constructing PPI. Target proteins related to diabetes were analyzed by online STRING 11.0 (https://string-db.org/cgi/input?sessionId=biiGmvCwYzjy&input_page_show_search=on) to construct the PPI network [34, 35].

#### 2.4.4. Component-Target-Pathway Network

The relationships of component, target, and pathway were clarified utilizing Cytoscape 3.7.2.

### 2.5. Cell Culture

HepG2 cells were grown in Dulbecco's modified eagle's medium (DMEM) supplemented with 10% fetal bovine serum, 100 IU/mL penicillin, and 100 *µ*g/mL streptomycin at 37°C, 5% CO_2_ having 95% relative humidity atmosphere. The cells were seeded at a density of 1.0 × 10^4^ cells/well in a 96-well plate for 24 h, and then, the cells were treated with high concentrations of insulin (1 × 10^−5^) for 48 h. After the period, the cells were divided into four groups (control, model, DMBG, and HG group). The control and model group were treated DMEM and DMBG, and the HG group was treated metformin (100 *μ*g/mL) and EJP (100 *μ*g/mL). After 24 h, the glucose content was measured using a spectrophotometric microtiter plate reader at 520 nm, and IR-HepG2 cells were prepared.

### 2.6. RT-PCR Analysis

The IR-HepG2 cells were extracted for RT-PCR analysis. First, the total RNA of IR-HepG2 was extracted with RNA rapid extraction solution, after treated as extracts of concentrations ranging from 100 to 500 ng/L. Second, the RNA was reverse-transcribed through a PCR instrument. Third, RT-PCR was performed using the PCR amplification instrument. The primer sequences were as follows: H-ACTIN-S: CACCCAGCACAATGAAGATCAAGAT; H-ACTIN-A: CCAGTTTTTAAATCCTGAGTCAAGC; H-PIK3r1-S: GGAAGCAGCAACCGAAACAA; H-PIK3r1-A: TCGCCGTCCACCACTACAGA; H-AKT (1)-S: GCTCAGCCCACCCTTCAAG; H-AKT (1)-A: GCTGTCATCTTGGTCAGGTGGT; H-GSK3*β*-S: GTTAGCAGAGACAAGGACGGCA; H-GSK3*β*-A: GCAATACTTTCTTGATGGCGAC; H-MYC-S: CTCGACTACGACTCGGTGCA; H-MYC-A: CGGGTCGCAGATGAAACTCT.

## 3. Results

### 3.1. Compound-Target Network

**Figure 1:**
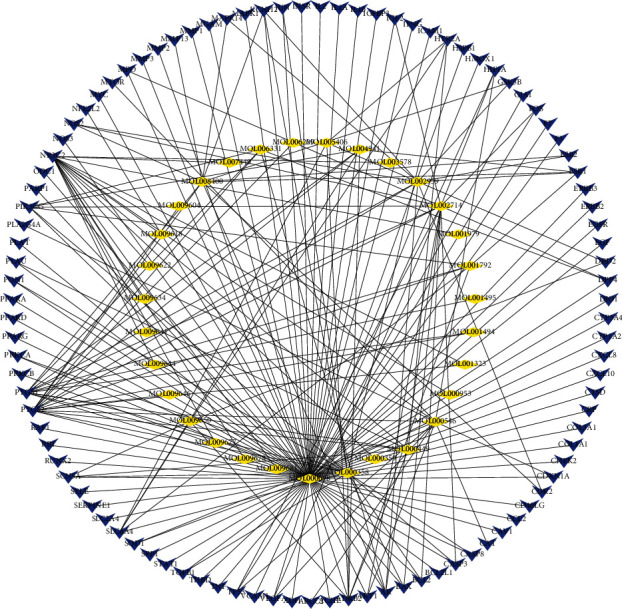
Components-target network was structured. The yellow 56 nodes represent the compounds; the blue 99 nodes represent the compound targets.

Ninety-nine (99) kinds of candidate genes relevant to diabetes were excavated from OMIM, GeneCards, Dis-GeNET, and TTD databases. As shown in Figure 1, 130 nodes (56 compound nodes and 99 target nodes) and 288 edges were seen in the compound-target network. The yellow and blue nodes represented the compounds and targets, respectively. Furthermore, each edge represented the correlation of compound and target. In the network, larger degree represents a stronger interaction. Furthermore, the larger degree nodes may represent the key compound or target in the network. Quercetin, *β*-sitosterol, diosgenin, baicalein, and 3′-methoxydaidzein were found as the potential composition of treating diabetes in EJP. Quercetin was connected to 85 proteins, *β*-sitosterol was associated with 15 proteins, diosgenin was related to 12 proteins, baicalein was relevant in 16 proteins, and 3′-methoxydaidzein was connected to 10 proteins.

### 3.2. GO and KEGG Analyses

**Figure 2:**
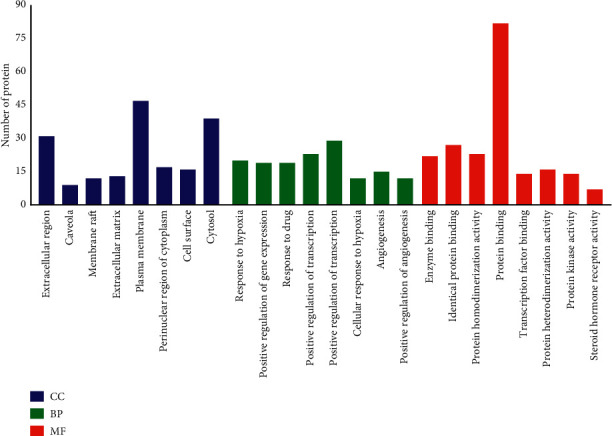
GO analysis, the distribution of GO entries in CC, BP, and MF (top 8 according to FDR <0.05) are shown.

**Figure 3:**
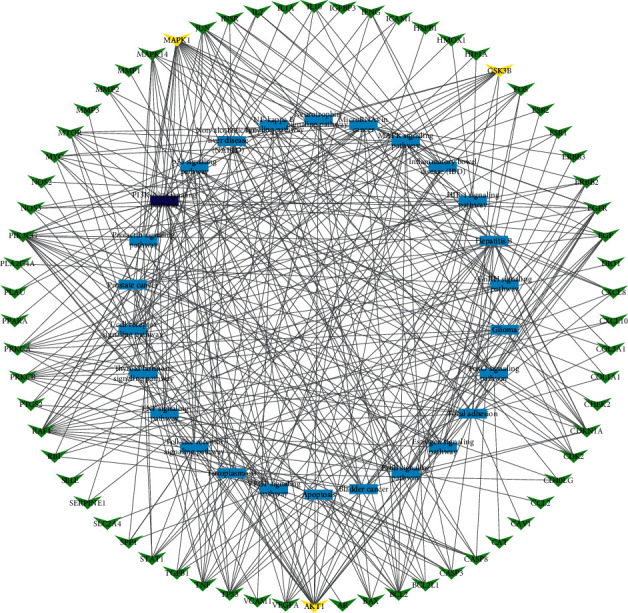
Target-pathway network constructed based on KEGG analysis from the DAVID database. Blue represents 26 pathways and green represents 66 protein targets.

**Figure 4:**
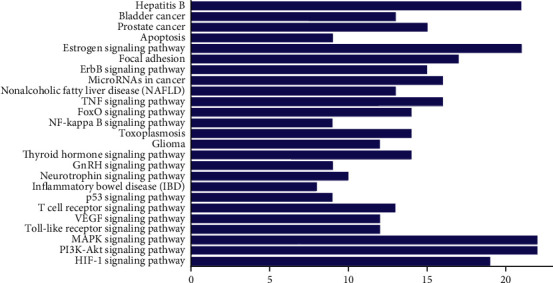
KEGG enrichment analysis of predicted pathways.

The DAVID 6.8 database (https://david.ncifcrf.gov) was utilized to elucidate the CC, BP, and MF annotations of the selected 99 proteins. There were 606 genes entries (FDR, Figure 2 shows the top 8 according to FDR <0.05), out of which 46 genes entries were relevant to CC including the extracellular region, plasma membrane, and cytosol. 477 items were related to BP, including the hypoxia and drug responsion, positive regulation of gene expression and transcription, DNA-templated, angiogenesis, inflammatory response, and positive regulation of cell proliferation. 83 items were related to MF, including protein binding, identical protein binding, and the homodimerization activity of protein. Therefore, the positive regulation of transcription with RNA polymerase II promoter, DNA-templated, and responsion of hypoxia about the action of EJP were regulated through the binding of protein, identical protein, and enzyme, as well as plasma membrane, cytosol, and the homodimerization activity of protein. A target-pathway network was constructed from the data screened of DAVID for determining the relationship between T2DM proteins and related pathways. Furthermore, the key signal pathways (HIF-1, PI3K-AKT, and MAPK) were based on the KEGG analysis (FDR <0.05) and literature analysis (Figures 3 and 4).

### 3.3. PPI Network Analysis

**Figure 5:**
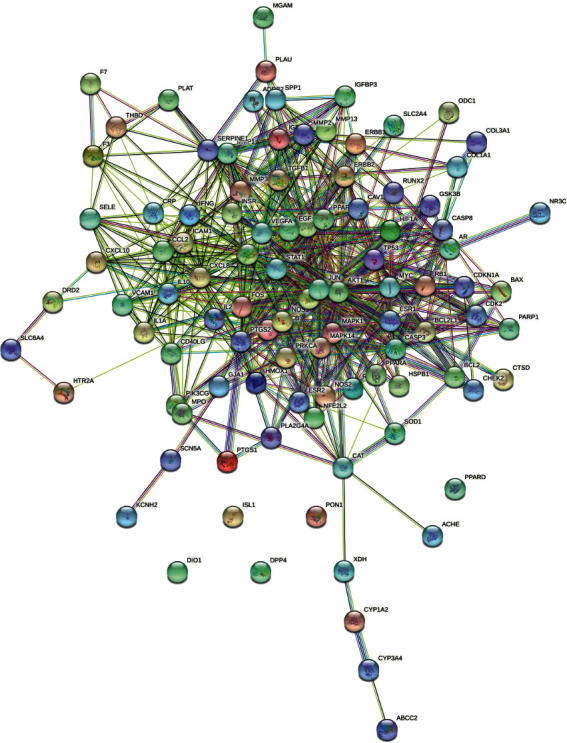
The network of protein-protein interaction.

The network of protein and protein was constructed for exploring the interaction of 99 antidiabetic protein. As shown in Figure 5, AKT1, EGF, EGFR, MAPK1, and GSK3*β* proteins were located at the core position. These five proteins mainly involved PI3K/AKT, MAPK, and VEGF signaling pathways. AKT1 regulated many processes including metabolism, proliferation, cell survival, growth, and angiogenesis. AKT1 was indirectly activated by insulin and other growth factors [36]. Inhibition of EGFR or HB-EGF intercepted the proliferative response to HB-EGF and glucose in rat islets [37]. MiR-133 may be an effective target for the treatment of diabetic nephropathy via the MAPK/ERK pathway [38]. The antidiabetic effect of SJE might be dependent on the AMPK pathway, which was indicated through the inhibition of gene expression in INS1 and GSK3*β*, and upregulating the hepatic phosphorylation of AMPK*α* in liver of mice [39].

### 3.4. Component-Target Pathway Network

**Figure 6:**
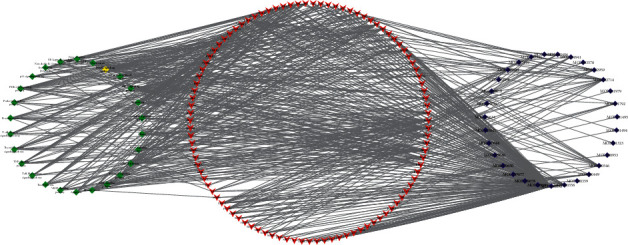
Component-target-pathway network was structured. From left to right, the components of EJP, target, pathways, and component represent the green, red, and blue, severally. The edges show the relationship of the component-target-pathway.

To further clarify the mechanism of action of EJP in the treatment of T2DM, the component-target-pathway network was established. As shown in Figure 6, the network was composed of chemical components, protein targets, and pathways, including 156 nodes and 643 edges. 56 components interacted with 99 target proteins and were associated with 26 pathways. Only 66 proteins were related to 26 pathways, so these 66 proteins were potential key proteins of EJP for T2DM. This prediction provided a scientific evidence for further research into the mechanism of EJP in the T2DM treatment.

### 3.5. RT-PCR Analysis

**Figure 7:**
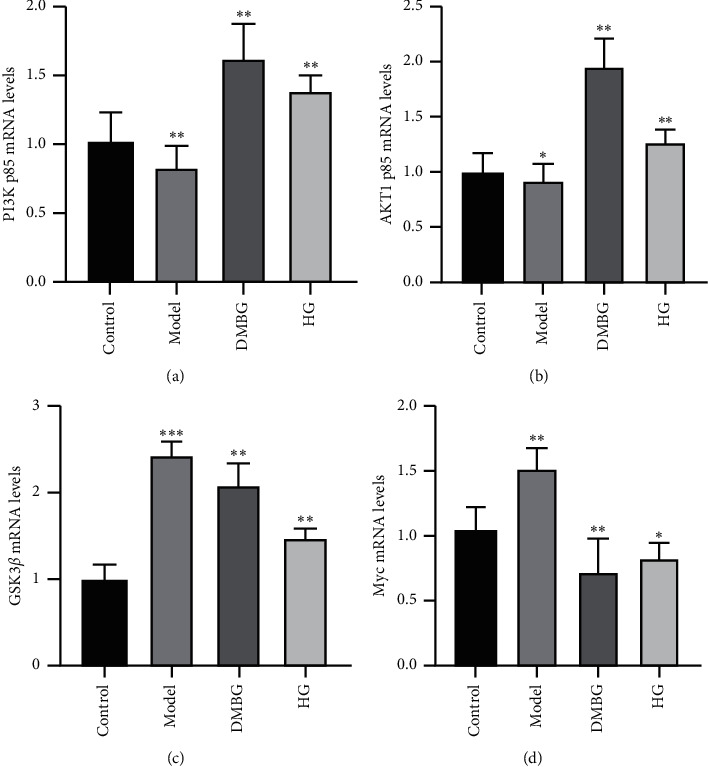
The RT-PCR analysis of the PI3K-AKT signal pathway. The expression level of PI3K p85, AKT1, GSK3*β*, and Myc mRNA. ^*∗*^*P* < 0.05. ^*∗∗*^*P* < 0.01.

Based on the prediction of network pharmacology, PI3K p85, AKT1, GSK3*β*, and Myc proteins in the PI3K-AKT signal pathway were selected and used to verify the mechanism of action of EJP in treating T2DM at the mRNA level by RT-PCR. As shown in Figure 7, compared to the control group, the expression level of the model group of PI3K p85 and AKT1 mRNA reduced while GSK3*β* and Myc mRNA increased. The expression level of DMBG and HG groups of PI3K p85 and AKT1 mRNA increased while GSK3*β* and Myc mRNA reduced compared with the model group. Thus, EJP controlled the blood glucose levels of T2DM mice via upregulating mRNA of PI3K p85 and AKT1 and downregulating mRNA of GSK3*β* and Myc in PI3K-AKT signal pathways.

## 4. Discussion

The prevalence rate of T2DM is gradually increasing due to the aging of the population and changes in lifestyle [40]. Long-term medication is inevitable for the treatment of such chronic metabolic disease. Due to TCM featured multitarget, multicomponent, and low toxicity became more and more popular in the treatment of diabetes. So, it is vital to elucidate the pharmacological mechanism of TCM formula. The active ingredient and targets of TCM could be preferentially predicted by network pharmacology [41]. In this study, the bioactive component and the action mechanism of EJP for diabetes treatment was predicted via the network pharmacology method, and some proteins were verified through RT-PCR monitoring.

The compound-target network clarified that the treatment efficacy of EJP against T2DM was exactly related 56 compounds. Quercetin, *β*-sitosterol, diosgenin, baicalein, and 3′-methoxydaidzein were found as the potential composition of treating diabetes in EJP. The hyperglycemia was modulated through regulating the enzymes activities as for metabolism in glucose and improving the antioxidant status of pancreatic in rats of T2DM model from the results of Oyedemi' studies [42]. Quercetin can be used for the treatment of T2DM by lowering the pancreatic iron deposition and PBC ferroptosis [43]. *β*-Sitosterol attenuates insulin resistance and high fat diet-induced detrimental changes via mediating IRS-1/AKT signaling for the management of T2DM [44]. Diosgenin ameliorates cognitive deficits in T2DM, owing to its amelioration of astrogliosis, inflammation, and oxidative stress [45]. Baicalein (10^−6^ and 10^−5^ mol/L) regulated glucose uptake, glycolysis, and gluconeogenesis of hepatocytes to treat T2DM [46]. 3′-Methoxydaidzein has not been reported about the antidiabetic effect and may be as a potential T2DM drug. Therefore, T2DM treated by EJP was closely related to key target compound including quercetin, *β*-sitosterol, diosgenin, baicalein, and 3′-methoxydaidzein. The network pharmacology prediction of EJP for the T2DM treatment has been proved to be appropriate, and the screening of new compounds for the T2DM treatment provides ideas for the development of new drugs.

The component-target-pathway network depicted that the therapeutic effect of EJP on T2DM directly interacted with 99 genes. The results of KEGG pathway enrichment analysis of 66 proteins indicated that 26 pathways were exactly connected to the occurrence and progression of T2DM, suggesting that these pathways might be the molecular mechanism of EJP against T2DM. The relationships of some pathways with T2DM were succinctly discussed as follows. Hepatitis B: A study showed hepatitis B virus (HBV) coinfection was significantly related to blood glucose levels. The participants of 28% with HBV coinfection developed T2DM. It was an increasing evidence that infection of HBV is strongly associated with the development of T2DM [47]. Bladder and prostate cancer pathway: T2DM are becoming increasingly prevalent worldwide and is associated with the increased incidence of bladder cancer [48]. However, a personal history of T2DM is connected with a lower incidence of prostate cancer [49]. Apoptosis pathway: apoptosis plays important roles in the pathophysiology of T2DM. The prevention and revert of *β*-cell apoptosis by regulating the balance of Bcl family, and apoptotic genes against apoptosis might be a new path for prevention and therapeutic application on T2DM [50]. FoxO signaling pathway: forkhead box protein O1 (FOXO1) played important roles in *β*-cell growth and function. FOXO1 mRNA levels were increased in the islets of patients with T2DM [51]. TNF signaling pathway: there was a growing evidence that tumor necrosis factor-*α* (TNF-*α*) involved in insulin resistance, and it is associated with the development of T2DM [52]. Glioma pathway: it was inverse relations between diabetes and glioma risk [53]. Thyroid hormone signaling pathway: the downregulation of FT3 was significantly related to the prevalence of DR in T2DM with normal thyroid function [54, 55]. Neurotrophin signaling pathway: the changes in the serum neurotrophic factor levels were associated with metabolic syndrome components in T2DM [56, 57]. T cell signaling pathway: in a study, it showed that the presence of senescent T cells exerted a detrimental influence on immune function during T2DM [58]. VEGF signaling pathway: the redox environment influences vascular endothelial growth factor (VEGF) production in response to proinflammatory stimuli in T2DM [59]. Toll-like receptor signaling pathway: toll-like receptor, the central of innate immunity, was significantly involved in progression of T2DM [60]. MAPK and PI3K-AKT signaling pathways: MAPK and PI3K-AKT are essential for glucose homeostasis. The PI3K-AKT signaling pathway is the major effector of metabolic insulin action [61, 62]. HIF-1 signaling pathway: hypoxia inducible factors (HIFs) play a value in T2DM. Furthermore, the HIF pathway is disordered in major metabolic tissues involved in the pathogenesis of diabetes [63]. According to the PPI network and RT-PCR analysis, PI3K-AKT, MAPK, and VEGF signaling pathways might play significant roles on T2DM.

## 5. Conclusion

99 targets and 58 signal pathways were screened via network pharmacology. Only 66 targets and 26 pathways were directly related to diabetes with literature analysis. In the compound-target network, quercetin, *β*-sitosterol, diosgenin, baicalein, and 3′-methoxydaidzein were core compounds. Furthermore, PI3K-AKT, MARK, and estrogen signal pathways might be significant. The expression of PI3K p85 and AKT1 p85 mRNA were upregulated and GSK3*β* and Myc mRNA were downregulated in the analysis of RT-PCR. It implied that the hyperglycemia of T2DM model rats were alleviated via the PI3K-AKT pathway. In conclusion, EJP was proved to treat T2DM. It is helpful for researchers to further design pharmacodynamics experiments on these components in the next protocol. Meanwhile, it provided a reference to study the effective constituents of EJP and mechanism of treating T2DM. These specific targets were also worth researching deeply in future.

## Figures and Tables

**Table 1:** Compounds database of two herbs in Erjing prescription.

MOL ID	Molecule	MW	OB (%)	DL	Medicine
MOL001323	Sitosterol *α*1	426.8	43.28	0.78	*L. barbarum*
MOL003578	Cycloartenol	426.8	38.69	0.78	*L. barbarum*
MOL001494	Mandenol	308.56	42	0.19	*L. barbarum*
MOL001495	Ethyl linolenate	306.54	46.1	0.2	*L. barbarum*
MOL001979	LAN	426.8	42.12	0.75	*L. barbarum*
MOL000449	Stigmasterol	412.77	43.83	0.76	*L. barbarum*
MOL005406	Atropine	289.41	45.97	0.19	*L. barbarum*
MOL005438	Campesterol	400.76	37.58	0.71	*L. barbarum*
MOL006209	Cyanin	411.66	47.42	0.76	*L. barbarum*
MOL007449	24-Methylidenelophenol	412.77	44.19	0.75	*L. barbarum*
MOL008173	Daucosterol_qt	414.79	36.91	0.75	*L. barbarum*
MOL008400	Glycitein	284.28	50.48	0.24	*L. barbarum*
MOL010234	*δ*-Carotene	536.96	31.8	0.55	*L. barbarum*
MOL000953	CLR	386.73	37.87	0.68	*L. barbarum*
MOL009604	14b-Pregnane	288.57	34.78	0.34	*L. barbarum*
MOL009612	(24*R*)-4*α*-Methyl-24-ethylcholesta-7, 25-dien-3*β*-yl acetate	482.87	46.36	0.84	*L. barbarum*
MOL009615	24-Methylenecycloartan-3*β*, 21-diol	456.83	37.32	0.8	*L. barbarum*
MOL009617	24-Ethylcholest-22-enol	414.79	37.09	0.75	*L. barbarum*
MOL009618	24-Ethylcholesta-5, 22-dienol	412.77	43.83	0.76	*L. barbarum*
MOL009620	24-Methyl-31-norlanost-9 (11)-enol	428.82	38	0.75	*L. barbarum*
MOL009621	24-Methylenelanost-8-enol	440.83	42.37	0.77	*L. barbarum*
MOL009622	Fucosterol	412.77	43.78	0.76	*L. barbarum*
MOL009631	31-Norcyclolaudenol	440.83	38.68	0.81	*L. barbarum*
MOL009633	31-Norlanost-9 (11)-enol	414.79	38.35	0.72	*L. barbarum*
MOL009634	31-Norlanosterol	412.77	42.2	0.73	*L. barbarum*
MOL009635	4, 24-Methyllophenol	414.79	37.83	0.75	*L. barbarum*
MOL009639	Lophenol	400.76	38.13	0.71	*L. barbarum*
MOL009640	4*α*, 14*α*, 24-Trimethylcholesta-8, 24-dienol	426.8	38.91	0.76	*L. barbarum*
MOL009641	4*α*, 24-Dimethylcholesta-7, 24-dienol	412.77	42.65	0.75	*L. barbarum*
MOL009642	4*α*-Methyl-24-ethylcholesta-7, 24-dienol	426.8	42.3	0.78	*L. barbarum*
MOL009644	6-Fluoroindole-7-dehydrocholesterol	402.7	43.73	0.72	*L. barbarum*
MOL009646	7-O-Methylluteolin-6-C-*β*-glucoside	318.3	40.77	0.3	*L. barbarum*
MOL009650	Atropine	289.41	42.16	0.19	*L. barbarum*
MOL009651	Cryptoxanthin monoepoxide	568.96	46.95	0.56	*L. barbarum*
MOL009653	Cycloeucalenol	426.8	39.73	0.79	*L. barbarum*
MOL009656	(*E*, *E*)-1-Ethyl octadeca-3, 13-dienoate	308.56	42	0.19	*L. barbarum*
MOL009660	Methyl (1R, 4aS, 7R, 7aS)-4a, 7-dihydroxy-7-methyl-1-[(2S, 3R, 4S, 5S, 6R)-3, 4, 5-trihydroxy-6-(hydroxymethyl) oxan-2-yl] oxy-1, 5, 6, 7a-tetrahydrocyclopentapyran-4-carboxylate	406.43	39.43	0.47	*L. barbarum*
MOL009662	Lantadene A	552.87	38.68	0.57	*L. barbarum*
MOL009664	Physalin A	526.58	91.71	0.27	*L. barbarum*
MOL009665	Physcion-8-O-*β*-D-gentiobioside	608.6	43.9	0.62	*L. barbarum*
MOL009677	Lanost-8-en-3*β*-ol	428.82	34.23	0.74	*L. barbarum*
MOL009678	Lanost-8-enol	428.82	34.23	0.74	*L. barbarum*
MOL009681	Obtusifoliol	426.8	42.55	0.76	*L. barbarum*
MOL000098	Quercetin	302.25	46.43	0.28	*L. barbarum*
MOL001792	DFV	256.27	32.76	0.18	*L. barbarum*
MOL002714	Baicalein	270.25	33.52	0.21	*L. barbarum*
MOL002959	3′-Methoxydaidzein	284.28	48.57	0.24	*L. barbarum*
MOL000358	*β*-Sitosterol	414.79	36.91	0.75	*L. barbarum/P. sibiricum*
MOL000359	Sitosterol	414.79	36.91	0.75	*P. sibiricum*
MOL003889	Methylprotodioscin_qt	446.74	35.12	0.86	*P. sibiricum*
MOL004941	(2*R*)-7-Hydroxy-2-(4 hydroxyphenyl) chroman-4-one	256.27	71.12	0.18	*P. sibiricum*
MOL000546	Diosgenin	414.69	80.88	0.81	*P. sibiricum*
MOL006331	4′, 5-Dihydroxyflavone	254.25	48.55	0.19	*P. sibiricum*
MOL009760	Sibiricoside A	432.71	35.26	0.86	*P. sibiricum*
MOL009763	(+)-Syringaresinol-O-*β*-D-glucoside	580.64	43.35	0.77	*P. sibiricum*
MOL009766	Zhonghualiaoine 1	458.75	34.72	0.78	*P. sibiricum*

**Table 2:** 99 genes related to compounds of EJP and T2DM.

Symbol name	Gene name	Symbol name	Gene name	Symbol name	Gene name
Prostaglandin G/H synthase 2	PTGS2	Acetylcholinesterase	ACHE	Mitogen-activated protein kinase 14	MAPK1
Prostaglandin G/H synthase 1	PTGS1	Epidermal growth factor receptor	EGFR	Interstitial collagenase	MMP1
Mineralocorticoid receptor	NR3C2	RAC-*α* serine/threonine-protein kinase	AKT1	Hypoxia-inducible factor 1-*α*	HIF1A
*β*-2 Adrenergic receptor	ADRB2	Vascular endothelial growth factor A	VEGFA	Signal transducer and activator of transcription 1-*α*/*β*	STAT1
Urokinase-type plasminogen activator	PLAU	Apoptosis regulator Bcl-2	BCL2	Receptor tyrosine-protein kinase erbB-2	ERBB2
Sodium channel protein type 5 subunit *α*	SCN5A	Bcl-2-like protein 1	BCL2L1	Peroxisome proliferator-activated receptor gamma	PPARG
5-Hydroxytryptamine 2A receptor	HTR2A	Proto-oncogene c-Fos	FOS	Heme oxygenase 1	HMOX1
Sodium-dependent serotonin transporter	SLC6A4	Cyclin-dependent kinase inhibitor 1	CDKN1A	Cytochrome P450 3A4	CYP3A4
D (2) dopamine receptor	DRD2	Apoptosis regulator BAX	BAX	Cytochrome P450 1A2	CYP1A2
Estrogen receptor	ESR1	72 kDa type IV collagenase	MMP2	Caveolin-1	CAV1
Androgen receptor	AR	Interleukin-10	IL10	Myc proto-oncogene protein	MYC
Peroxisome proliferator-activated receptor-*γ*	KCNH2	Proepidermal growth factor	EGF	Tissue factor	F3
Estrogen receptor *β*	ESR2	Retinoblastoma-associated protein	RB1	Gap junction *α*-1 protein	GJA1
Mitogen-activated protein kinase 14	MAPK14	Tumor necrosis factor	TNF	Intercellular adhesion molecule 1	ICAM1
Glycogen synthase kinase-3 *β*	GSK3*β*	Transcription factor AP-1	JUN	C-C motif chemokine 2	CCL2
Cell division protein kinase 2	CDK2	Caspase-3	CASP3	E-selectin	SELE
Nitric oxide synthase, inducible	NOS2	Cellular tumor antigen p53	TP53	Vascular cell adhesion protein 1	VCAM1
Collagenase 3	MMP13	Ornithine decarboxylase	ODC1	Interleukin-8	CXCL8
Phosphatidylinositol-4, 5-bisphosphate 3-kinase catalytic subunit, *γ* isoform	PIK3CG	Xanthine dehydrogenase/oxidase	XDH	Protein kinase C *β* type	PRKCB
Dipeptidyl peptidase IV	DPP4	Caspase-8	CASP8	Heat shock protein *β*-1	HSPB1
Stromelysin-1	MMP3	RAF proto-oncogene serine/threonine-protein kinase	RAF1	Transforming growth factor *β*-1	TGFB1
Coagulation factor VII	F7	Superoxide dismutase (Cu-Zn)	SOD1	Maltase-glucoamylase, intestinal	MGAM
Nitric oxide synthase, endothelial	NOS3	Protein kinase C alpha type	PRKCA	Interleukin-2	IL2
Tissue-type plasminogen activator	PLAT	Interferon gamma	IFNG	Poly (ADP-ribose) polymerase 1	PARP1
Thrombomodulin	THBD	Interleukin-1*α*	IL1A	Solute carrier family 2, facilitated glucose transporter member 4	SLC2A4
Plasminogen activator inhibitor 1	SERPINE1	Myeloperoxidase	MPO	Collagen *α*-1 (III) chain	COL3A1
Collagen *α*-1 (I) chain	COL1A1	Nuclear factor erythroid 2-related factor 2	NFE2L2	Serine/threonine-protein kinase Chk2	CHEK2
C-reactive protein	CRP	C-X-C motif chemokine 10	CXCL10	Osteopontin	SPP1
Runt-related transcription factor 2	RUNX2	Cathepsin D	CTSD	Insulin-like growth factor-binding protein 3	IGFBP3
Insulin-like growth factor II	IGF2	Serum paraoxonase/arylesterase 1	PON1	Cytosolic phospholipase A2	PLA2G4A
CD40 ligand	CD40LG	Type I iodothyronine deiodinase	DIO1	Canalicular multispecific organic anion transporter 1	ABCC2
Receptor tyrosine-protein kinase erbB-3	ERBB3	Catalase	CAT	Serine/threonine-protein kinase mTOR	MTOR
Insulin receptor	INSR	Peroxisome proliferator-activated receptor-*α*	PPARA	Peroxisome proliferator-activated receptor *δ*	PPARD

## Data Availability

The data used to support the findings of this study are included within the article.

## Abbreviations

EJP: Erjing prescription
T2DM: Type 2 diabetes mellitus
RT-PCR: Real-time PCR
CC: Cell component
BP: Biological process
MF: Molecular function
PPI: Protein-protein interactions
TCM: Traditional Chinese medicine
TCMSP: Traditional Chinese medicine systems pharmacology
OB: Oral bioavailability
DL: Drug-likeness
GO: Gene Ontology
TTD: Therapeutic target database
KEGG: Kyoto Encyclopedia of Genes and Genomes.

## Supplementary Materials

Supplementary Materials The graphical abstract is the overall flowchart of this study and may be found online in the supporting materials section at the end of the article.
