# Supplementary material for: A Network Pharmacology Approach to Investigate the Mechanism of Erjing Prescription in Type 2 Diabetes
Source: Evid Based Complement Alternat Med. 2021 Jul 24;2021:9933236. doi: 10.1155/2021/9933236 (PMC8328705; doi:10.1155/2021/9933236)
Supplement: Supplementary Materials — The graphical abstract is the overall flowchart of this study and may be found online in the supporting materials section at the end of the article. [file 9933236.f1.pdf]

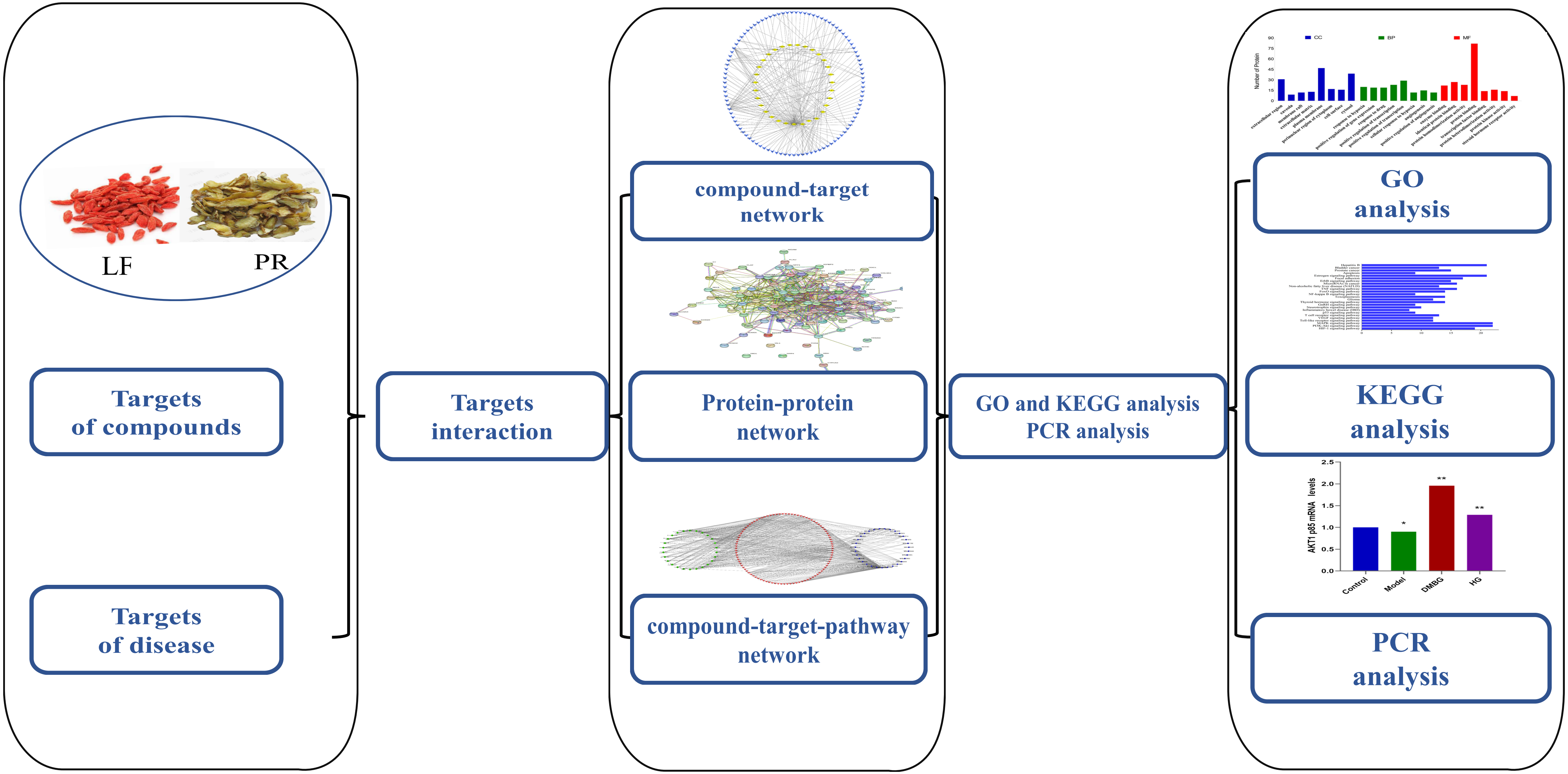
LF
PR
Targets of compounds
Targets of disease
Targets interaction
compound-target network
Protein-protein network
compound-target-pathway network
GO and KEGG analysis PCR analysis
CC
BP
MF
GO analysis
KEGG analysis
AKT1 p85 mRNA levels
Control
Model
DMBG
HG
PCR analysis
